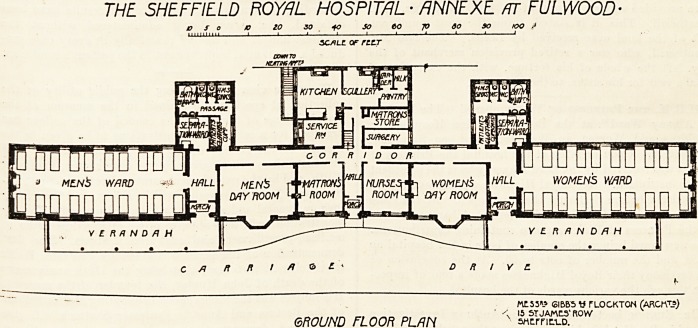# Sheffield Royal Hospital Annexe at Fulwood

**Published:** 1908-10-17

**Authors:** 


### HOSPITAL ADMINISTRATION.

### CONSTRUCTION AND ECONOMICS.

### SHEFFIELD ROYAL HOSPITAL ANNEXE AT FULWOOD.

This valuable annexe to the Sheffield Hospital is not a convalescent home in the ordinary acceptation of the term, but is intended for the reception of patients who have been under treatment in the hospital itself, and who might have for one reason or other to be discharged from the wards ere the necessity for medical attendance and careful nursing were over. Beyond doubt such an institution might play a most important part in the treatment of a large number of cases, especially when the parent hospital is situated in a crowded city.

The annexe owes its existence to the munificence of Mr. Meggitt Johnson, M.P., who provided all the money required for the building; and it was formally opened a few months since by Mrs. Johnson in presence of a large company, which included the Lord Mayor and Lady Mayoress of Sheffield, the Bishop of Sheffield, Sir John Bingham, Mr. Wake, Chairman of the Hospital Board, etc., etc.

For the equipment and furnishing the annexe is indebted to Mr. Jackson Smith and Mr. Philip Wake.

The site is an extensive one, being not less than 11 acres, and it has the advantage of commanding fine views of the Whiteley Woods and the hills beyond them. The site was bought by the Governors of the hospital, and it cost £5,000, which sum, however, included the laying out of the grounds.

The annexe consists of an administrative centre and two wings. The latter are of one story only, and the former is of two stories. The ground floor of the centre contains the men's day room and the women's day room, having between them the matron's room, the nurses' room, and the entrance hall. Over this part of the administrative block are six rooms for the staff, and behind the ground-floor rooms a corridor runs the entire length of the block, and gives access to the kitchen department, which is placed at the back; and at each end the corridor opens into a lobby or hall, with separate entrance, from which lobby spring the ward pavilions. At the junction of the corridor and lobby is the separation ward of one bed only, and the sanitary annexe; in fact, the separation ward and the annexe really constitute one block. The various parts of the sanitary annexe are well enough arranged, and are properly cut off from the main building by cross-ventilated passages; but the single-bedded separation wards have, so far as we can see, no cross ventilation at all, which omission we look upon as a mistake. The men's pavilion is placed at right angles to the block just described. The ward is about 60 feet long and 20 feet wide; and as it contains twenty beds, each bed will have a floor space of 60 square feet, and, assuming a ceiling 13 feet high, there will be cubic space of about 780 feet per bed. This is not an excessive allowance; but, of course, much depends on the class of patients for which the annexe is intended, and also on the means which may be in use to ensure thorough ventilation.

The beds, excepting the end ones, are arranged in pairs, and so each pair has a window on both sides, and not each bed, as is the rule in modern hospital construction; but here again the remark made as to cubic space might apply. The women's ward is a replica of the men's ward, and each has a fine verandah running almost the entire length of the ward.

In general conception the plan is good; but it resembles a cottage hospital rather than an annexe to a large infirmary. The cardinal point is not given, but we should imagine that the front elevation of the building faces south, and, if so, one aspect of the wards must be north.

The architects were Messrs. Gibbs and Flockton, of Sheffield; the contractors for the joinery were Messrs. Badger and Sons; for the masonry, Mr. Henry Turton; and for laying out the grounds, Messrs. Hadfield and Son. The cost of the hospital, exclusive of land and furniture, was £5,000, the whole of which was given by Mr. Johnson.

## Figures and Tables

**Figure f1:**